# Effects of RAF inhibitors on PI3K/AKT signalling depend on mutational status of the RAS/RAF signalling axis

**DOI:** 10.18632/oncotarget.6959

**Published:** 2016-01-20

**Authors:** Raphaela Fritsche-Guenther, Franziska Witzel, Stefan Kempa, Tilman Brummer, Christine Sers, Nils Blüthgen

**Affiliations:** ^1^ Max-Delbrück-Center for Molecular Medicin (MDC) Berlin Buch, The Berlin Institute for Medical Systems Biology (BIMSB), 13125 Berlin, Germany; ^2^ Institute of Pathology, Molecular Tumor Pathology, Charité - Universitätsmedizin Berlin, 10117 Berlin, Germany; ^3^ Institute for Theoretical Biology, Charité - Universitätsmedizin Berlin, 10115 Berlin, Germany; ^4^ Institute of Molecular Medicine and Cell Research and Centre for Biological Signalling Studies *BIOSS*, Albert-Ludwigs University Freiburg, 79104 Freiburg, Germany; ^5^ Integrative Research Institute for the Life Sciences, Humboldt University Berlin, 10099 Berlin, Germany

**Keywords:** sorafenib, signal transduction networks, BRAF, KRAS, colon cancer

## Abstract

Targeted therapies within the RAS/RAF/MEK/ERK signalling axis become increasingly popular, yet cross-talk and feedbacks in the signalling network lead to unexpected effects. Here we look systematically into how inhibiting RAF and MEK with clinically relevant inhibitors result in changes in PI3K/AKT activation. We measure the signalling response using a bead-based ELISA, and use a panel of three cell lines, and isogenic cell lines that express mutant forms of the oncogenes *KRAS* and *BRAF* to interrogate the effects of the MEK and RAF inhibitors on signalling. We find that treatment with the RAF inhibitors have opposing effects on AKT phosphorylation depending on the mutational status of two important oncogenes, *KRAS* and *BRAF*. If these two genes are in wildtype configuration, RAF inhibitors reduce AKT phosphorylation. In contrast, if *BRAF* or *KRAS* are mutant, RAF inhibitors will leave AKT phosphorylation unaffected or lead to an increase of AKT phosphorylation. Down-regulation of phospho-AKT by RAF inhibitors also extends to downstream transcription factors, and correlates with apoptosis induction. Our results show that oncogenes rewire signalling such that targeted therapies can have opposing effects on parallel pathways, which depend on the mutational status of the cell.

## INTRODUCTION

During the past decade, AKT has emerged as a central player in signal transduction pathways activated in response to insuline or growth factors. The activated kinase AKT regulates a number of targets, with more than 100 AKT substrates known today [1–3]. The majority of the substrates is involved in essential biological functions such as proliferation, survival and apoptosis, and AKT has also been implicated in epithelial to mesenchymal transition [4, 2, 3]. The signalling pathway involving the activation of PI3K that leads to AKT activation is well studied, and many growth factors trigger this pathway. In addition to the PI3K/AKT pathway, growth factors also activate the mitogenic RAS/ERK cascade. Thus, cell survival (PI3K/AKT) and mitogenic (RAS/ERK) cascades rarely act as independent parallel pathways. Rather, they influence each other at different points and phases of signal transduction in positive and negative direction, resulting in a dynamic and complex cross-talk. For example, perturbation of MEK increases EGFR induced AKT activation [5–7]. Similarly, strong IGF stimulation results in an activation of AKT which in turn cross-inhibits ERK signalling. Mechanistically, this is thought to be mediated by phosphorylating RAF at inhibitory sites by AKT [8–12]. These cross-talk mechanisms prevent ERK-dependent growth arrest and promote proliferation [9].

The significant advances in elucidating these pathways have resulted in a better understanding of tumors that are driven by these pathways, and an improved survival. One example is colorectal cancer, where 50% of patients carry a mutant KRAS in their tumors indicating that the other half of patients could respond to anti EGFR therapy [13]. Nevertheless, 40% of patients with wildtype KRAS do not respond. In these patients mutant BRAF, which is present in 5–10% of the tumors, may affect response outcome [13, 14]. Beside KRAS and BRAF mutations, about 40% of malignant tumors carry known activating PI3K/AKT alterations [15], emphasising the importance to understand the signalling between the two pathways. Information about the tumor genotype, and in particular about mutations in PI3K/AKT, KRAS and BRAF are currently used to predict the success of systemic chemotherapy of different types of tumors and are used to stratify patients for treatment options for example in colon cancer [16].

The multikinase inhibitor Sorafenib was initially designed as a CRAF inhibitor, but is also a potent inhibitor of BRAF. The inhibitor has been shown to induce apoptosis and necrosis in various types of tumors cells, e.g. in acute myeloid leukaemia, renal cell and hepatocellular carcinoma [17–21]. Sorafenib has been shown to modulate AKT activity. In hepatocellular carcinoma cells (HCC), Sorafenib treatment leads to activation of AKT and up-regulation of its downstream factors [22, 23]. The increase in phosphorylated AKT and the existing cross-talk between the PI3K/AKT and RAS/ERK axis indicate a compensatory mechanisms of the PI3K/AKT pathway which may contribute to Sorafenib resistance [24, 25]. However, the mechanisms behind this cross-talk remain unclear.

In this study we have identified an interaction between RAF and AKT pathways, in which inhibition of RAF with Sorafenib and other RAF inibitors negatively regulates AKT activity resulting in increased apoptosis and reduction of proliferation. Interestingly, this effect is only apparent in cells harbouring no mutations in either KRAS or BRAF. While the mechanism behind this cross- talk remains unclear, the response of AKT to Sorafenib is predicted to be a good marker for therapeutic efficiency of Sorafenib, as it correlates both with genotype and phenotype after treatment.

## RESULTS

### Decreased AKT phosphorylation in RAS/RAF wildtype colon carcinoma cells after RAF inhibition

**Figure 1:**
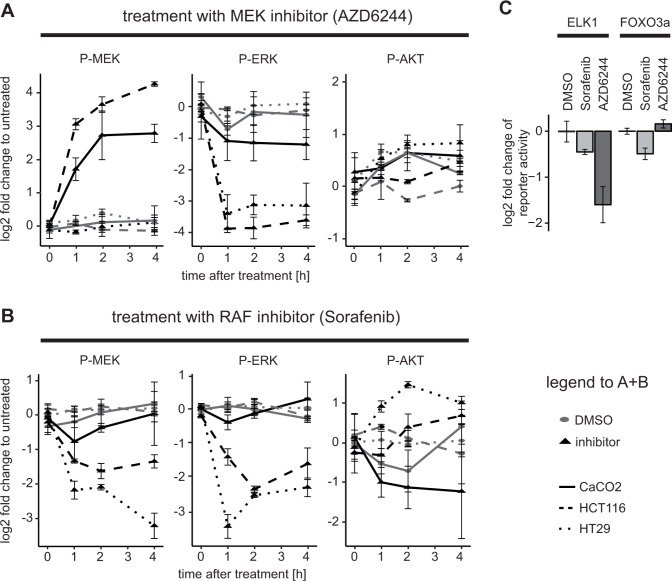
Downregulation of AKT activity and downstream targets after application of the RAF inhibitor Sorafenib in KRAS/BRAF wildtype cells (**A** and **B**) HCT116, HT29 and CaCO2 cells were treated with (A) 1 μM AZD6244 or (B) 10 μM Sorafenib, or their solvent control (DMSO) for the time durations indicated and signalling was measured using a bead-based ELISA (Luminex platform) with *n* ≥ 3 replicates. Mean and standard deviations are shown. (**C**) CaCO2 cells were transfected with 300 ng ELK and 20 ng Renilla or 300 ng FOXO3a and 20 ng Renilla luciferase reporter constructs for 24 h and then treated with 10 μM Sorafenib, 1 μM AZD6244 or DMSO for 4 h.

The network of RAS/ERK and PI3K/AKT signalling pathway is a complex regulatory circuit, which includes feedback loops and cross-talk between the two pathways. Importantly, these feedbacks and cross-talk can shape the response of tumor cells to targeted therapies, which may be different depending on mutations in the pathway, such as in *KRAS* or *BRAF* genes [26, 6, 27]. To explore how different mutations change the response of tumor cells to drugs we treated CaCO2 (wildtype for *BRAF* and *KRAS*), HCT116 (*KRAS^G13D^*), and HT29 (*BRAF^V600E^*) colon carcinoma cells with the MEK inhibitor AZD6244 or with the RAF inhibitor Sorafenib for various periods of time. We then assayed pathway activity by measuring phospho-AKT, phospho-MEK and phospho-ERK using bead-based ELISAs (Luminex). As shown in Figure 1A (left panel) we found that MEK phosphorylation was increased upon treatment with MEK inhibitors in CaCO2 and HCT116, and was unchanged in HT29 cells. This finding is in line with previous reports that attribute this effect to a feedback loop from ERK to RAF, which induces RAF activity and MEK phosphorylation when cells are treated with a MEK inhibitor [26, 28]. When cells depend on a *BRAF^V600E^* mutation like HT29 cells, this feedback is disrupted and thus MEK phosphorylation is not increased [26, 27]. When we treated the cells with the RAF inhibitor Sorafenib, we observed no increase in MEK phosporylation, but a decrease in most cell lines (Figure 1B, left panel), confirming that Sorafenib does block RAF activity. When we monitored AKT activity, we saw a modest increase of phospho-AKT both after treatment with MEK inhibitor and Sorafenib in HCT116 and HT29 (Figure 1A and 1B, right panel). Also this increase confirms previous reports that inhibition of MAPK signalling sensitises the EGF receptor and thereby induces AKT [6]. Unexpectedly, however, we found a decrease in AKT activation in CaCO2 cells, when they were treated with Sorafenib (Figure 1B, right panel).

To investigate whether these rather surprising effects of Sorafenib on AKT signalling in CaCO2 cells manifests itself also on downstream processes, we performed reporter assays for two transcription factors, ELK1 and FOXO3a, which are downstream of ERK and AKT, respectively. In agreement with the signalling data, ELK1 activity was down-regulated both by the RAF inhibitor Sorafenib and the MEK inhibitor treatment, albeit MEK inhibition resulted in more pronounced reduction of ELK activity (Figure 1C). The FOXO3a reporter showed reduced activity post Sorafenib treatment, and a mild up-regulation after treatment with the MEK inhibitor (Figure 1C). Thus, these experiments confirm that the effects of Sorafenib on signalling also extend to transcription factors downstream of ERK and AKT.

**Figure 2:**
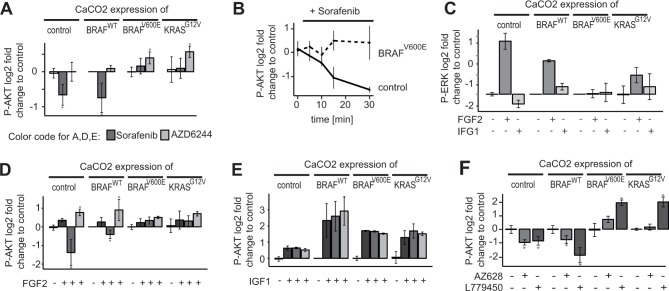
Downregulation of AKT activity by Sorafenib is restricted to BRAF/KRAS wildtype cells (**A**) CaCO2 control cells and CaCO2 cells expressing wildtype, V600E mutated BRAF or G12V mutated KRAS were treated with 10 μM Sorafenib, 1 μM AZD6244 or PBS for 4 h. Signalling was measured using multiplex assays (Luminex platform). After Sorafenib treatment, phospho-AKT is lower compared to PBS in CaCO2 cells expressing the control vector or wildtype BRAF, but remains unchanged in cells expressing BRAF^V600E^ or KRAS^G12V^. AKT-phosphorylation is either not affected or increased in all cell lines when treated with AZD6244. (**B**) CaCO2 control and cells expressing V600E mutated BRAF were treated with 10 μM of Sorafenib for times indicated. Phospho-AKT is down-regulated in a time-dependent manner in control cells, but not in cells expressing BRAF^*V600E*^. (**C**) CaCO2 control cells and cells expressing wildtype, V600E mutated BRAF or G12V mutated KRAS were stimulated with 0.005 μg/ml FGF2, 0.1 μg/ml IGF1 or PBS as a control (**D**) CaCO2 control cells and cells expressing wildtype, V600E mutated BRAF or G12V mutated KRAS were treated with 10 μM Sorafenib, 1 μM AZD6244 or PBS for 2 h following stimulation with 0.005 μg/ml FGF2. AKT phosphorylation is lower compared to PBS in cells expressing wildtype BRAF or control vector, but not in cells harboring a BRAF^*V600E*^ or KRAS^*G12V*^ mutation. Phospho-AKT is increased in all cells when treated with AZD6244. (**E**) CaCO2 control cells and cells expressing wildtype, V600E mutated BRAF or G12V mutated KRAS were treated with 10 μM Sorafenib, 1 μM AZD6244 or PBS for 2 h following stimulation with 0.1 μg/ml IGF1. AKT phosphorylation increases independently of MEK or RAF inhibition. (**F**) CaCO2 control cells and cells expressing wildtype, V600E mutated BRAF or G12V mutated KRAS were treated with 200 nM AZ628 and 10 μM L779450. All data (*n* ≥ 3 replicates) were measured with Luminex technology and shown as log2 fold change and standard deviation. Significant deviations are indicated with asterisk (*p* < 0.05).

We observed that Sorafenib inhibited AKT activity only in CaCO2 colon carcinoma cells that are BRAF and KRAS wildtype, and led to an increase in AKT activity in the other cell lines, which had mutations in either KRAS or BRAF. We therefore hypothesised that Sorafenib mediated inhibition of AKT signalling only occurs if the RAS/RAF signalling axis is wildtype. To test this, we used CaCO2 cells, which were stably transfected with inducible BRAF^*WT*^, mutant BRAF^*V600E*^ or KRAS^*G12V*^, and an empty expression vector as control. Figure 2A shows that indeed Sorafenib treatment resulted in the down-regulation of activated AKT in control cells and cells over-expressing wildtype BRAF. In contrast, those cells over-expressing KRAS^*G12V*^ or BRAF^*V600E*^ showed unchanged AKT phosphorylation. One could hypothesise that the effect of Sorafenib is mediated by MAPK signalling, however, the experiments with the MEK inhibitor AZD6244 showed that MEK inhibition does not change AKT phosphorylation in KRAS/BRAF wildtype cells.

To get further insights if Sorafenib treatment affects AKT phosphorylation only as a secondary adaptive response of the cells, i.e. that the cells are in a different state and express many other genes, or that the cells have a different cell-cycle distribution, we analysed the kinetics by which Sorafenib reduces AKT activity. CaCO2 and CaCO2 cells expressing BRAF^*V600E*^ were treated with Sorafenib for various time points between 10 min and 30 min. As shown in Figure 2B, phospho- AKT was down- regulated in CaCO2 cells at early time points (10–15 min), which indicates that the effect is not mediated by secondary responses such as transcriptional alterations.

Next, we wanted to investigate how RAF inhibition modulates the response of AKT to external stimuli. We decided to use two growth factors, FGF2 and IGF1, that are known to activate preferentially the RAF/MEK/ERK and PI3K/AKT signalling axes, respectively. We stimulated CaCO2 cells harbouring the empty vector control, or overexpressing wildtype BRAF, BRAF^*V600E*^ and KRAS^*G12V*^. We observed that FGF2 primarily stimulated ERK phosphorylation, and triggers a mild increase in AKT phosphorylation (Figure 2C, 2D) in all four cell lines. In contrast, IGF1 treatment alone had little effects on ERK and led to activation of AKT in these cell lines (Figure 2C, 2E).

To study the effect of RAF and MAPK signalling on AKT, we then pre-incubated the cells for 2 h with RAF or MEK inhibitors before we stimulated them. Again, we found that Sorafenib led to a decrease of AKT signalling in BRAF/KRAS wildtype cells, even when cells were stimulated with FGF2 (Figure 2D), and Sorafenib had no effect on AKT in cells expressing mutant BRAF or KRAS. Pre-incubation with the MEK inhibitor AZD6244 led to an increase of AKT phosphorylation in most cell lines when compared to treatment with FGF2 alone. This is in line with previous results that show sensitisation of receptors due to negative feedback, albeit sensitisation was most pronounced for the EGFR [6]. Interestingly, we observed that Sorafenib and AZD6244 had no effect on AKT signalling triggered by IGF1 (Figure 2E), suggesting that Sorafenib does not affect the IGF/PI3K/AKT core signalling module.

Sorafenib is known to have a wide range of targets apart from RAF [21], and therefore the observed effects on AKT may be due to other targets apart from RAF. We therefore tested whether other RAF inhibitors have similar effects on RAF. We treated our panel of CaCO2 control cells, and CaCO2 cells expressing wildtype BRAF, and mutant BRAF and KRAS with two additional inhibitors of RAF (AZ628, L779450) and measured the response of AKT, ERK and MEK phosphorylation (Figure 2F, Supplementary Figure 1). We find that both inhibitors elicit a similar response to Sorafenib, as they block MEK/ERK signalling irrespective of the mutation, but only lead to a down-regulation of phospho-AKT signalling, when BRAF and KRAS are wildtype.

Taken together, the results so far indicate that RAF inhibitors modulate AKT signalling response to external cues that also trigger MAPK signalling. It has opposite effects on AKT in KRAS/BRAF wildtype versus mutated cells, it affects response to external stimuli, but it does not modulate the core PI3K/AKT module.

### RAF-inhibitor-induced AKT inhibition extends to other cell types and leads to an inhibition of proliferation and induced apoptosis

**Figure 3:**
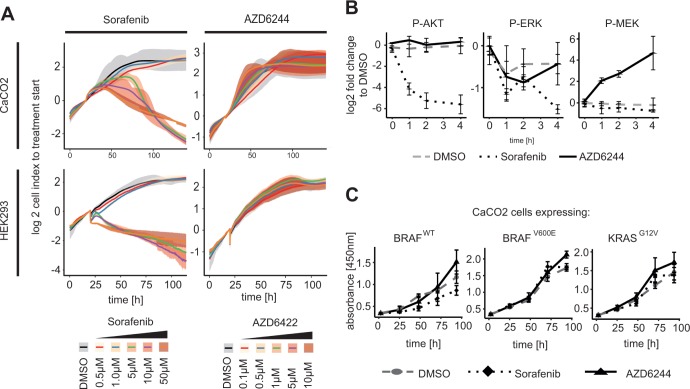
RAF inhibitor induced AKT phosphorylation leads to inhibition of proliferation in KRAS/BRAF wildtype cells (**A**) Cell index of a real-time cell proliferation (XCelligence RTCA) of CaCO2 and HEK293 cells in response to concentrations indicated of Sorafenib or AZD6244 compared to solvent control DMSO was measured up to 120 h. Proliferation was significantly reduced at 5 μM to 50 μM Sorafenib, while proliferation was unaffected using MEK inhibitor. Mean value of cell index and spread of *n* = 3 replicates were shown. (**B**) HEK cells were treated with 10 μM Sorafenib, 1 μM AZD6244 or DMSO for indicated times. Decreased AKT activity was detected in cells treated with Sorafenib, while not when perturbed with AZD6244. (**C**) CaCO2 cells expressing wildtype, V600E mutated BRAF or G12V mutated KRAS were treated with 10 μM Sorafenib, 1 μM AZD6244 or DMSO for 24 h up to 96 h and analyzed with XTT for proliferation activity. Proliferation decreased significantly in wildtype BRAF after Sorafenib treatment, but not in cells expressing BRAF^*V*600*E*^ or KRAS^*G*12*V*^. Mean value and standard deviation of *n* = 3 replicates is shown.

Based on the observation that RAF inhibition down-regulates AKT activity we were keen to understand whether the reduction of AKT affects proliferation or apoptosis. We measured CaCO2 cell growth using real-time RTCA technology [29] (Figure 3A). We observed decreased proliferation in CaCO2 cells after 24 h exposure with different concentrations of Sorafenib (Figure 3A upper left panel). In contrast, following treatment with the MEK inhibitor AZD6244, proliferation remained unaffected over time independent of inhibitor concentration (Figure 3A upper right panel).

We were interested if reduced proliferation due to Sorafenib treatment is restricted to colon cancer cell lines, or whether it extends to other cell types. We therefore measured proliferation in human embryonic kidney (HEK) cells. We found that also this cell line showed strong reduction in growth when treated with Sorafenib, but no growth reduction when treated with the MEK inhibitor (Figure 3A lower panel). This raised the question whether the RAF-inhibitor-induced AKT inhibition is a general mechanism that occurs when KRAS or BRAF are wildtype. To investigate this, we also treated HEK cells with RAF inhibitor Sorafenib and AZD6244 up to 4 h and measured signalling. We observed a response similar to the one in CaCO2 cells described above: AZD6244 treatment resulted in unchanged AKT activity, decreased phospho-ERK level and, possibly due to feedback regulation, increased MEK phosphorylation (Figure 3B). Sorafenib treatment again resulted in reduced AKT phosphorylation, reduced MEK and ERK signalling. This shows that the effect of Sorafenib on AKT is not limited to colon cancer cell lines, but also extends to other cell types with wildtype RAS/RAF-signalling axis such as HEK cells.

We then set out to investigate whether the effect of Sorafenib on proliferation is dependent on a wildtype RAS/RAF/ERK signalling axis, by using the isogenic CaCO2 cell lines that express inducible BRAF^*WT*^, BRAF^*V600E*^ or KRAS^*G12V*^. We treated these cells with Sorafenib or AZD6244 and measured proliferation using the metabolic XTT assay. We observed that cell proliferation was significantly decreased after Sorafenib treatment in CaCO2 cells that expressed BRAF^*WT*^, while it was unchanged when mutant BRAF or KRAS were expressed (Figure 3C).

**Figure 4:**
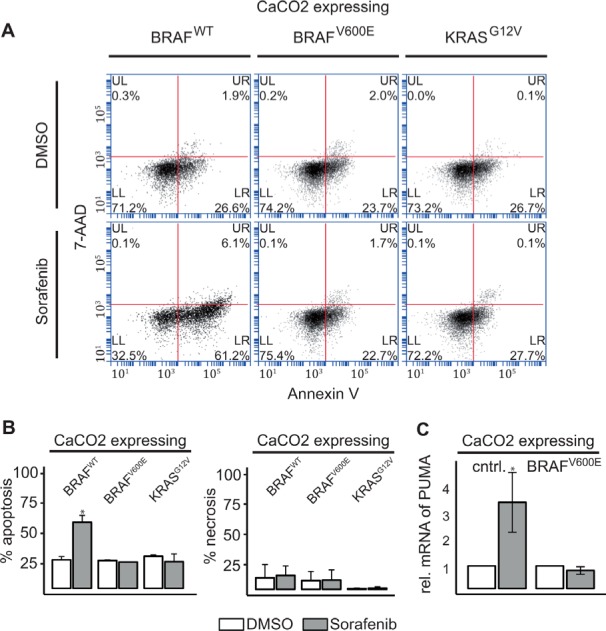
RAF inhibitor induced AKT phosphorylation leads to induction of apoptosis in KRAS/BRAF wildtype cells (**A**) Apoptotic (Annexin V:PE+ and 7−AAD−) and necrotic cells (Annexin V:PE+ and 7−AAD+) were analyzed by flow cytometry 48 h after treatment with 10 μM Sorafenib or DMSO in CaCO2 cells expressing wildtype, V600E mutated BRAF or G12V mutated KRAS. (**B**) The bars show the % of measured apoptosis or necrosis from *n* = 2 replicates. CaCO2 cells expressing wildtype BRAF showed an increase in apoptosis compared to BRAF^*V*600*E*^ or KRAS^*G*12*V*^ mutated cells. Necrosis was unaffected by treatment with Sorafenib after 48 h. (**C**) Relative expression of *PUMA* mRNA measured using qRT-PCR 48 h after treatment of control cells and CaCO2 cells expressing V600E mutated BRAF with 10 μM Sorafenib compared to DMSO treatment. *PUMA* was upregulated in wildtype CaCO2 cells. Mean and standard deviation are for *n* = 3 replicates. Significant deviations are indicated with asterisk (*p* < 0.05).

We then assessed whether apoptosis and necrosis was induced by Sorafenib in these cells, by measuring phycoerythrin (PE) labeled Annexin V and 7-Amino-actinomycin D staining (See Figure 4A). Sorafenib treatment significantly induced apoptosis after 48 h in cells expressing BRAF^*WT*^, but not in those expressing BRAF^*V600E*^ or KRAS^*G12V*^ (See Figure 4A, 4B). Interestingly, Sorafenib treatment did not affect necrosis (Figure 4A, 4B). In line with increased apoptosis, PUMA (BBC3), a member of the BCL-2 family of pro-apoptotic proteins and direct target of FOXO3a [30], is significantly up-regulated in CaCO2 cells compared to BRAF^*V600E*^-expressing cells after Sorafenib treatment for 48 h (Figure 4C).

## DISCUSSION

Mutations in the KRAS and BRAF genes are thought to drive many tumors, and the signalling cascades and downstream effectors with regard to those oncogenes are considered as attractive pharmacological targets [31]. Nevertheless, the effects of known and unknown feedbacks and cross-talk are complicating the effort to design effective compounds for patient specific therapy [6, 27, 32, 33]. In this regard, cross-modulation of the PI3K/AKT pathway, which controls processes like cell survival and growth, has been implicated in drug resistance [6, 33]. Reports about interaction between RAF/MEK/ERK signalling and PI3K/AKT are manifold. For instance, strong IGF stimulation results in a cross inhibition of RAF by AKT. In that case, AKT negatively regulates ERK activation by the phosphorylation of a CRAF inhibitory site (Serine 259) [8–12]. Such cross-talk seem to depend on the type of ligand and the cellular background or stage of differentiation [9].

In this study, we systematically interrogated the effect of inhibiting MEK and RAF on both MAPK signalling and AKT signalling in various colon cancer cell lines with and without mutations in BRAF and KRAS. We focussed our study on colorectal carcinoma cell lines, because approximately 50% of colorectal tumors contain a mutated KRAS gene [15], and further 10% harbor mutated BRAF [15, 34].

Many effects of the inhibitors on upstream signalling and AKT signalling that we observed confirm previous results and can be attributed to feedbacks. For example, MEK inhibition leads to higher MEK phosphorylation in BRAF wildtype cells, which can be attributed to a feedback from ERK to RAF [26–28]. Another example is the activation of AKT after MEK inhibition in BRAF/KRAS mutant cell lines, which has been reported to be mediated by a relieve of feedback inhibition of the EGF receptor after drug treatment [33, 6, 5, 7, 35]. In contrast, we found a rather surprising effect of Sorafenib (Nexavar) on AKT signalling: In cells that have a wildtype KRAS/RAF/MEK/ERK signalling axis, Sorafenib treatment reduces AKT signalling, while in cells with mutant KRAS or BRAF, AKT signalling tends to be up-regulated after Sorafenib treatment. Using two additional structurally unrelated RAF inhibitors we confirmed that the observed effect is not specific to Sorafenib, but rather an effect of blocking RAF. Reduced AKT signalling after Sorafenib treatment in KRAS/BRAF wildtype cells also extends to downstream target levels, as shown with reduced FOXO3a activity after Sorafenib treatment. We furthermore show that the effects on AKT activity are indepenent of MAPK signalling, as MEK inhibition does not result in downregulation of AKT phosphorylation.

Very importantly and in line with the role of AKT as a survival pathway, we find an induction of cell death as evidenced by cleavage of caspase 3 and the nuclear protein PARP. Induction of apoptosis after treatment with Sorafenib was shown for several types of cancer [17, 36, 37, 18, 38], but the mechanism behind the induction of apoptosis remain unclear, and has so far been attributed to its effects on ERK signalling, and not been linked to reduced AKT phosphorylation [39, 40, 20].

Several studies in leukaemia cell lines have shown results which are in line with our study. For example, induction of apoptosis and decrease of AKT activation occurred in acute lymphoblastic leukemia (ALL) cells after treatment with Sorafenib [41, 42]. Importantly, the used ALL cell lines were wildtype for KRAS and BRAF mutations. Other leukemic cell lines such as MV4–11, KG1 or OCI/AML3, which are also wildtype for BRAF or KRAS, showed an elevated apoptosis and decreased proliferation after treatment with Sorafenib [17, 38]. A similar pattern was observed in the lymphoma cell line SUD-DHL-4 V, which does not harbor mutations in KRAS and BRAF. Contrary, RAS mutated Hodgkin, pancreatic or hepatocellular carcinoma cell lines showed increased P-AKT after perturbation with Sorafenib [43]. Taken together, these studies underline the consequence of KRAS mutations on AKT activation after treatment with Sorafenib. In pancreatic cells harboring a KRAS^*G*12*V*^ mutation, no change in P-AKT occurs after inhibition of RAF, with only a mild increase in apoptosis. Very interestingly, in cells with a KRAS^*Q*61*H*^ mutation, a decrease in P-AKT level and a high increase in apoptosis were shown, which was comparable to wildtype cells [44]. These observations suggest that that distinct KRAS mutants differ in their spectrum of pathway crosstalk.

Taken together our results show strongly different, even opposing response of AKT signalling in wildtype compared to KRAS and BRAF mutated cells after RAF inhibition.

## MATERIALS AND METHODS

### Cells and cell culture

The cell lines HEK293, HCT116, HT29 and CaCO2 were obtained from ATCC (American Type Culture Collection, UK). These cell lines were maintained in DMEM (Dulbecco's Modified Eagle's Medium, Lonza) supplemented with 10% fetal calf serum, 1% ultraglutamine and 1% penicillin/streptomycin. All cells were incubated in a humidified atmosphere of 5% CO_2_ in air at 37 grad celcius. Caco2tet cells and their derivatives Caco2tet/empty vector, Caco2tet/BRAF^*WT*^, Caco2tet/BRAF^*V600E*^ and Caco2tet/KRAS^*G12V*^, have been described previously [26, 45]. The doxycyclin inducible expression system is described in detail elsewhere [46].

### Reagents

The following inhibitors were used in the various assays: MEK inhibitor AZD6244 (1 μM unless otherwise specified; Selleck Chemicals) and RAF inhibitor Sorafenib (10 μM unless otherwise specified, LC Laboratories), AZ628 (200 nM, Selleck Chemicals) and L779450 (10 μM, Abcam). The solvent control was the appropriate amount of DMSO. We used the following ligands (all Peprotech): IGF1 (0.1 μg/ml), and FGF2 (0.005 μg/ml) solved in 0.01% BSA (bovine serum albumin) in PBS (phosphate buffered saline).

### Luciferase reporter gene assay

CaCO2 cells were co-transfected with luciferase expression vector encoding FOXO3a (AddGene) or ELK1 (kind gift of Markus Morkel, Charite) and Renilla luciferase vector (internal control) using Lipofectamine 2000 (Life Technologies). After 4.5 h medium was replaced and Sorafenib or AZD6244 was added for 4 h. Cells were harvested with passive cell lysis buffer (Promega), and luciferase activity was measured using the dual luciferase reporter assay system (Promega) according to the manufacturer's instructions. The relative activity was normalised to the ratio of Firefly luciferase activity to Renilla luciferase activity and calculated as the fold difference from treatment to untreated control for each expression vector.

### Luminex bead-based technology

After treatment of cells lysates were collected and the level of phospho-protein expression was analysed with the Luminex system (BioRad, Hercules, CA) using beads specific for phospho-MEK1 (S217/S221), phospho-AKT (S473) and phospho-ERK2 (Thr185/Tyr187) according to the manufacturer's instructions. Briefly, samples were washed with PBS and lysed with cell lysis buffer (BioRad). Lysate protein concentration was determined with BCA (bicinchoninic acid) method (Thermo Scientific). The beads and detection antibodies were diluted 1:3. For acquiring data, the BioPlex Manager software was used.

### RNA isolation and quantitative RT-PCR analysis

RNA was isolated from cells after treatment with Sorafenib using the RNeasy-mini-kit (Qiagen) according to the suppliers protocol. Quantitative real-time PCR analysis was performed using a StepOnePlus 96-well format Light-Cycler apparatus (Applied Biosystems). Experiments were run and analysed with the StepOne 2.0 software according to the manufacturer's recommendations. Synthesis of double-stranded DNA during the PCR cycles was visualised with TaqMan gene expression assays FAM-dye labeled (PUMA [Hs_00248075_m1]) or VIC-dye labeled for loading control (PGK1 [Hs_943178_g1]) and TaqMan gene expression master mix (all Applied Biosystems). The data were analysed quantitatively by measuring the threshold cycles (CT).

### Proliferation studies

Metabolic activity was determined using the tetrazolinum salt XTT (Roche) according to the manufacturer's protocol. Briefly, cells were seeded in 96-well plates in triplicates and treated with Sorafenib, AZD6244 or DMSO. After time indicated, cells were incubated with XTT labeling mixture and measured using an ELISA reader (Benchmark Plus BioRad) at 480 nm with a reference wavelength at 680 nm. For proliferation studies in real time, the XCelligence RTCA SP instrument was used. CaCO2 and HEK cells were plated 24 h before treatment with inhibitors or controls. Over time of measurement, the system records the cell index (CI), which reflects the cell attachment to the electrodes and is proportional to the number of cells. Each treatment was measured at least in triplicates. As the initial cell number is variable and growth of the cell population is exponential, averaging the growth curves is not reasonable. Instead, we calculated the logarithmised and normalized cell index log2(ci(t)/ci(t of inhibitor application)) and took the average of these values [29].

### Analysis of apoptosis and necrosis

Apoptosis and necrosis was determined using Annexin V:PE and 7-AAD Apoptosis detection kit (BD Pharmingen) and flow cytometry analyses. In brief, cells were seeded and treated after 24 h with Sorafenib or DMSO. Cells were incubated for 48 h and harvested. Each cell pellet was resuspended in 100 μl of binding buffer and 5 μl of Annexin V:PE and 5 μl of 7-AAD were added. After incubation time of 15 min at room temperature, additional 100 μl of binding buffer were added. Flow cytometry analyses were performed using BD Accuri C6 flow cytometer and data thus obtained were analysed with FlowJo 10 software.

## SUPPLEMENTARY MATERIALS FIGURE


