# Supplementary material for: Effects of RAF inhibitors on PI3K/AKT signalling depend on mutational status of the RAS/RAF signalling axis
Source: Oncotarget. 2016 Jan 20;7(7):7960–9. doi: 10.18632/oncotarget.6959 (PMC4884967; doi:10.18632/oncotarget.6959)
Supplement: Supplementary file 1 [file oncotarget-07-7960-s001.pdf]

Effects of RAF inhibitors on PI3K/AKT signalling depend on mutational status of the RAS/RAF signalling axis

Supplementary Materials

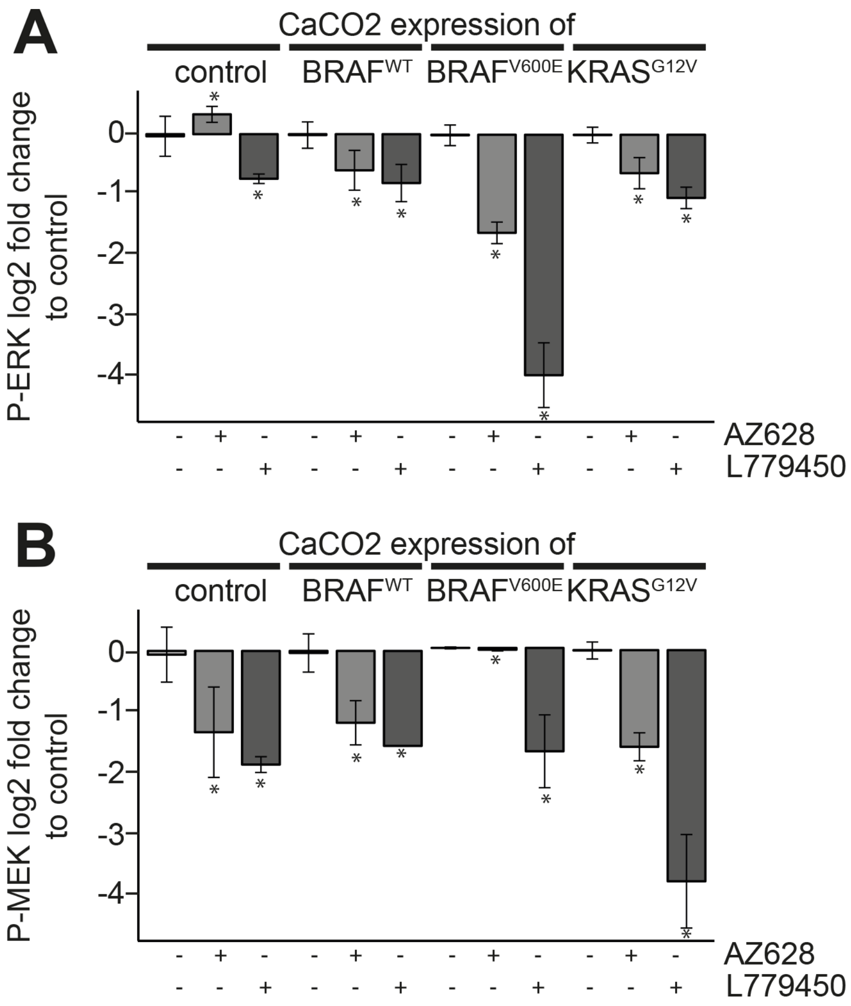

Supplementary Figure S1: CaCO2 control and cells expressing wildtype, V600E mutated BRAF or G12V mutated KRAS were treated with the RAF inhibitors AZ628 and L779450 for 4 h. Phospho-ERK and phospho-MEK were measured with Luminex technology and shown as log2 fold change compared to DMSO treatment ($n \geq 3$ replicates).
